# Preoperative Serum Sodium Level as a Prognostic and Predictive Biomarker for Adjuvant Therapy in Esophageal Cancer

**DOI:** 10.3389/fonc.2020.555714

**Published:** 2021-01-21

**Authors:** Qifeng Wang, Lin Peng, Yongtao Han, Tao Li, Wei Dai, Yi Wang, Lei Wu, Yang Wei, Tianpeng Xie, Qiang Fang, Qiang Li, Jinyi Lang, Bangrong Cao

**Affiliations:** ^1^Department of Radiation Oncology, Sichuan Cancer Hospital & Institute, Sichuan Cancer Center, School of Medicine, University of Electronic Science and Technology of China, Chengdu, China; ^2^Radiation Oncology Key Laboratory of Sichuan Province, Sichuan Cancer Hospital & Institute, Sichuan Cancer Center, School of Medicine, University of Electronic Science and Technology of China, Chengdu, China; ^3^Department of Thoracic Surgery, Sichuan Cancer Hospital & Institute, Sichuan Cancer Center, School of Medicine, University of Electronic Science and Technology of China, Chengdu, China; ^4^Department of medical oncology, Sichuan Cancer Hospital & Institute, Sichuan Cancer Center, School of Medicine, University of Electronic Science and Technology of China, Chengdu, China

**Keywords:** serum sodium levels, hyponatremia, inflammation, adjuvant therapy, biomarker

## Abstract

**Background:**

Low serum sodium has been associated with unfavorable outcome in several cancers. The prognostic value of serum sodium in esophageal carcinoma (EC) remains unclear. This study aimed to investigate preoperative serum sodium in association with outcome and survival benefit of adjuvant therapy for patients with EC.

**Methods:**

Preoperative serum sodium and clinicopathological indexes were retrospectively analyzed in 2155 patients who underwent esophagectomy at Sichuan cancer hospital. Overall survival (OS) and disease-free survival (DFS) were analyzed by using Kaplan-Meier method and Cox regression. Benefit of adjuvant therapy was estimated by using Propensity Score Matching.

**Results:**

The incidence of hyponatremia and hypernatremia were 2% (43/2155) and 3.5% (76/2155) in treatment-naive patients. Both sodium disorders indicated unfavorable OS (hyponatremia, adjusted HR[95% CI] = 1.78[1.2–2.62]; hypernatremia, adjusted HR = 1.52[1.1–2.11]) and DFS (hyponatremia, adjusted HR[95% CI] = 1.52[1.03–2.23]; hypernatremia, adjusted HR = 1.45[1.06–1.99]). Decreased sodium concentrations within the normal range were associated with poor OS and DFS. Postoperative adjuvant therapy was associated with improved three-year OS (56.6 vs. 40%; adjusted HR = 0.55 [95% CI, 0.41–0.73]) and DFS (51.9 vs. 36.2%; adjusted HR = 0.63 [95% CI, 0.48–0.83]) versus surgery alone in patients with low serum sodium (Na < 139.6 mmol/liter), but not in other sodium subgroups. Meanwhile, serum sodium was inversely correlated with cell counts of leukocytes, neutrophils, monocytes and C-reactive protein levels.

**Conclusions:**

These results suggested that low preoperative serum sodium is associated with poor outcome in EC patients, and may predict survival benefit of adjuvant therapy.

## Introduction

Esophageal cancer (EC) is the ninth most common malignancy worldwide, which accounts for proximately 508,585 cancer-related deaths (1). Squamous-cell carcinoma (SCC) is the predominant histological type of EC, especially in Asian countries (2). The 5-year overall survival of EC patients ranged from 15–25% (2). Patients with locally advanced diseases are generally treated with a multimodal approach which includes various combinations of surgery, chemotherapy and radiation (2). Postoperative chemotherapy or chemoradiation could improve outcome in patients with high-risk factors, such as locally advanced disease, lymph node involvement or incomplete resection (3–9). However, little attention has been paid to identify biomarkers that could guide adjuvant therapy for EC patients.

Hyponatremia is a frequent electrolyte disorder in patients with cancer. The reported incidence of hyponatremia ranged from 1–76% across different cancer types (10). Hyponatremia was associated with poor prognosis of patients with cancers in lung, colon and rectal, breast, liver, kidney, ovary, as well as head and neck (11–16). The incidence and prognostic value of hyponatremia in EC patients remain underestimated. In previous studies, hyponatremia was reported as an adverse event in 16–59% of EC patients who had received chemotherapy or chemoradiation (17–22). However, hyponatremia induced by chemotherapy seemed not to be correlated with outcome of patients with EC (21). The incidence and prognostic association of hyponatremia in treatment-naïve EC patients remains unclear. Meanwhile, whether the preoperative serum sodium concentrations could serve as a predictive marker for adjuvant therapy is also unknown.

In the present study, we retrospectively reviewed 2,155 EC patients who underwent esophagectomy alone, and esophagectomy followed by adjuvant therapy (chemotherapy or chemoradiation). The preoperative serum sodium concentrations were analyzed in association with overall survival (OS) and disease free survival (DFS). To analyze the survival benefit of adjuvant therapy, a sub-cohort was generated by Propensity Score Matching (PSM). In addition, the associations of preoperative serum sodium levels with leukocytes, lymphocytes, neutrophils, monocytes as well as CRP levels were investigated.

## Materials and Methods

### Study Cohort

This study enrolled 2,155 patients who had received esophagectomy at Sichuan Cancer Hospital from January 2009 to August 2017. Restrospective data including clinical parameters, preoperative sodium concentrations, postoperative treatment options as well as follow-up information were collected from these patients. All patients were diagnosed by histological pathology flowing surgery resection. Pathological stage was re-defined according to the eighth edition of the AJCC TNM classification system (23). Exclusion criterions were as follows: (1) primary tumor *in situ* by pathology; (2) those who received neoadjuvant chemotherapy or chemoradiotherapy before surgery; (3) those who received palliative surgery following definitive radiation; (4) patients who lost follow-up information or other clinicopathological or laboratorial parameters. This study was approved by the Institutional Ethics Committee of Sichuan Cancer Hospital.

In the study population, 1,357 patients received surgery alone (Surgery), 798 patients received surgery followed by adjuvant chemotherapy (CT) or concurrent chemoradiotherapy (CCRT). Patients with traditional high-risk prognostic factors were usually received adjuvant CT/CCRT (eg, T3 or advanced stages, lymph node involved, R1/R2 resection, vascular invasion, neural invasion or poor histological differentiation), except for those with physical or other reasons. In the adjuvant subgroup, 588 patients received chemotherapy which was started at 4–6 weeks after surgery. The rest 210 patients received concurrent chemotherapy and Intensity Modulated Radiation Therapy (IMRT). Radiotherapy was delivered with a total dose of 50–54 Gy/25–30 fractions (5 fractions per week for 5–6 weeks). Chemo regimens included Cisplatin-, Nedaplatin-, Oxaliplatin-, and Carboplatin-based chemotherapies and Fluorouracil alone chemotherapy, which were delivered to 325, 192, 152, 14, and 115 patients, respectively. OS and DFS times were defined as the peroids from surgery treatment to patient death and locoregional and/or distant recurrence, respectively.

### Assessment of Serum Sodium and Other Blood Parameters

Preoperative serum sodium concentration was routinely assessed in the clinical laboratory at Sichuan cancer hospital. Hyponatremia and hypernatremia were defined by serum sodium levels < 135 and > 145 mmol/liter as previously described (16). Within the reference range of serum sodium level, the first to fourth quartiles were 135 to 139.6 mmol/liter, 139.6 to 141.05 mmol/liter, 141.05 to 142.4 mmol/liter, and 142.4 to 145 mmol/liter, respectively.

Other blood parameters including cell counts of total leukocytes, neutrophils, lymphocytes, monocytes were assessed at the same time with serum sodium. Two inflammatory response indexes, neutrophil to lymphocyte ratio (NLR) and lymphocyte to monocyte ratio (LMR) were calculated. In addition, C-reactive protein (CRP) levels in preoperative serum were available in 664 patients.

### Statistical Analysis

Patient and tumor characteristics were compared with subgroups stratified by preoperative serum sodium levels by using the Chi-squared test or Fisher’s exact test when appropriate. Continuous variables in accordance with normal distribution were compared by using *t*-test between two groups or One-way ANOVA among three or more groups. Mann-Whitney test or Kruskal-Wallis test were performed for variables that did not follow a normal distribution. Multiple tests were adjusted by using the Bonferroni method. OS and DFS were compared among sodium subgroups by using Kaplan-Meier curves and log-rank test. Univariate Cox regression analysis was performed for serum sodium and other confounders when appropriate, including sex, age at diagnosis, Karnofsky Performance Status (KPS) score, tumor histology, tumor location, tumor differentiation, resection margin, vascular invasion, neural invasion, dissected lymph node number, pTNM stage and treatment groups. Multivariable Cox regression was carried out by using variables with *p* value < 0.1 in the univariate analysis.

Survival benefit of adjuvant CT/CCRT compared with surgery alone was evaluated. To minimize confounding effects between the two treatment groups, a propensity-score matching (PSM) was performed. A 1:1 matched study cohort was created by using the variables of sex, age, KPS, histology pathology, tumor location, tumor differentiation, resection margin, vascular invasion, neural invasion, dissected number of lymph node and pTNM stage. In the matched cohort, OS and DFS were compared between S + CT/CCRT and surgery alone by using KM curves and Cox regression, which were stratified by serum sodium levels. All statistical computations were performed using R software v.3.5.1 (https://www.r-project.org/) and a *p* value (two-sided) of <0.05 was considered to be statistically significant.

## Results

### Patient Characteristics

Of all patients, 81.2% were male, 39% were 65 years in age or older, 38% of patients had KPS score of 70–80 (**Table 1**). The majority of patients had squamous cell histology (96.8%) and R0 resection (95.6%). 53.3% of cancers were located in the middle of the esophagus. There were 18.1, 20, and 9.1% of cancers with vascular invasion, neural invasion and dissected lymph node number less than 10, respectively. The median OS and DFS after initial surgical resection were 47.8 months (95% CI, 42.7–54.4 months) and 40.6 months (95% CI, 37.2–45.1 months). The median follow-up time for patients still alive was 33 months (interquartile range: 22–51.1 months).

**Table 1:** Characteristics of patients and cancers by serum sodium levels.

Variables	Levels	All Num (%)	Serum sodium			*p* value
			Hyponatremia Num (%)	Normal Num (%)	Hypernatremia Num (%)	
Age						0.583
	<65ys	1305(60.56)	29(67.4)	1232(60.5)	44(57.9)	
	≥65ys	850(39.44)	14(32.6)	804(39.5)	32(42.1)	
Sex						0.011
	Male	1749(81.16)	40(93)	1655(81.3)	54(71.1)	
	Female	406(18.84)	3(7)	381(18.7)	22(28.9)	
KPS						0.451
	90–100	1336(62)	28(65.1)	1256(61.7)	52(68.4)	
	70–80	819(38)	15(34.9)	780(38.3)	24(31.6)	
Tumor location						0.932
	upper	556(25.8)	13(30.2)	524(25.7)	19(25)	
	middle	1148(53.27)	20(46.5)	1087(53.4)	41(53.9)	
	lower	451(20.93)	10(23.3)	425(20.9)	16(21.1)	
Histology						*0.146
	SCC	2087(96.84)	39(90.7)	1974(97)	74(97.4)	
	ADC	17(0.79)	1(2.3)	16(0.8)	0(0)	
	Neur.C	18(0.84)	1(2.3)	17(0.8)	0(0)	
	others	33(1.53)	2(4.7)	29(1.4)	2(2.6)	
Differentiation						0.495
	G1	428(19.86)	10(23.3)	405(19.9)	13(17.1)	
	G2	855(39.68)	20(46.5)	808(39.7)	27(35.5)	
	G3	872(40.46)	13(30.2)	823(40.4)	36(47.4)	
Surgical margin						0.876
	R0	2060(95.59)	41(95.3)	1946(95.6)	73(96.1)	
	R1	69(3.2)	2(4.7)	65(3.2)	2(2.6)	
	R2	26(1.21)	0(0)	25(1.2)	1(1.3)	
Varscular invasion						0.221
	No	1766(81.95)	39(90.7)	1662(81.6)	65(85.5)	
	Yes	389(18.05)	4(9.3)	374(18.4)	11(14.5)	
Neural invasion						0.625
	No	1724(80)	35(81.4)	1625(79.8)	64(84.2)	
	Yes	431(20)	8(18.6)	411(20.2)	12(15.8)	
Dissected lymph node number						0.749
	≥10	1960(90.95)	38(88.4)	1854(91.1)	68(89.5)	
	<10	195(9.05)	5(11.6)	182(8.9)	8(10.5)	
pTNM						0.007
	IA	31(1.44)	1(2.3)	29(1.4)	1(1.3)	
	IB	205(9.51)	3(7)	196(9.6)	6(7.9)	
	IIA	350(16.24)	2(4.7)	339(16.7)	9(11.8)	
	IIB	361(16.75)	5(11.6)	345(16.9)	11(14.5)	
	IIIA	156(7.24)	1(2.3)	142(7)	13(17.1)	
	IIIB	794(36.84)	22(51.2)	742(36.4)	30(39.5)	
	IVA	252(11.69)	8(18.6)	238(11.7)	6(7.9)	
	IVB	6(0.28)	1(2.3)	5(0.2)	0(0)	
Adjuvant therapy						0.957
	No	1357(62.97)	28(65.1)	1281(62.9)	48(63.2)	
	Yes	798(37.03)	15(34.9)	755(37.1)	28(36.8)	
NLR						0.015
	<2.6	1071(49.7)	12(27.9)	1020(50.1)	39(51.3)	
	≥2.6	1084(50.3)	31(72.1)	1016(49.9)	37(48.7)	
LMR						<0.001
	<3.94	1072(49.74)	33(76.7)	1006(49.4)	33(43.4)	
	≥3.94	1083(50.26)	10(23.3)	1030(50.6)	43(56.6)	

ys, years; pTNM, pathological Tumor-Node-Metastasis staging; KPS, Karnofsky Performance Status; NLR, neutrophil-to-lymphocyte ratio; LMR, lymphocyte-to-monocyte ratio; Num, number; *, Fisher’s exact test; other p value, Chi-squared test. Serum sodium levels: Hyponatremia, <135 mmol/liter; Normal, 135–145 mmol/liter; Hypernatremia, >145 mmol/liter.

The median of preoperative serum sodium level was 141.1 mmol/liter (range: 115.9–164.1 mmol/liter). There were 2036 patients (94.5%) who had serum sodium within the reference range (135–145 mmol/liter). The incidence of hyponatremia and hypernatremia were 2% (43/2155) and 3.5% (76/2155) in whole cohort. The incidence of hyponatremia in patients with cancers of early stage (IA-IIB) and late stage (IIIA - IVB) were 1.2% (11/947) and 2.6% (32/1208) respectively. Furthermore, preoperative serum sodium levels showed significant association with gender (*p* = 0.01), pTNM stages (*p* = 0.007), NLR (*p* = 0.015), and LMR (*p* < 0.001), respectively (**Table 1**).

### Preoperative Hyponatremia and Hypernatremia as Unfavorable Prognostic Factors

**Figure 1:**
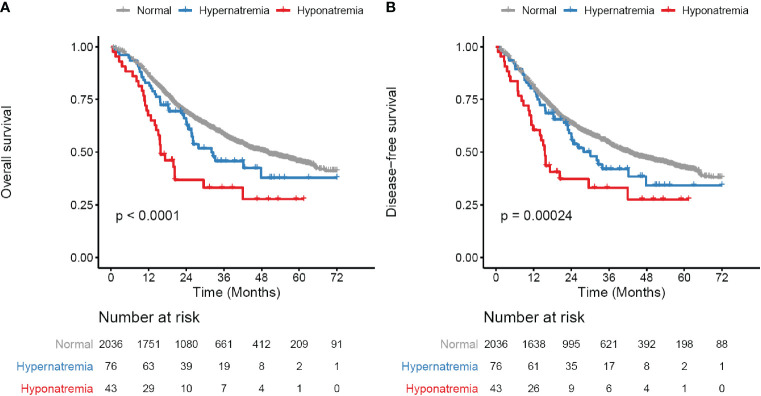
Survival curves for preoperative serum sodium in whole study cohort. **(A)** Overall survival (OS) curves for three subgroups defined by serum sodium levels. **(B)** Disease-free survival (DFS) curves for three subgroups defined by serum sodium levels. Serum sodium subgroups: normal, 135–145 mmol/liter; Hypernatremia, > 145 mmol/liter; Hyponatremia, < 135 mmol/liter. Survival curves are generated by using Kaplan-Meier methods. *p* value is calculated by log-rank test.

Patients with preoperative hyponatremia and hypernatremia had worse outcomes than those with normal serum sodium levels (OS and DFS, both *p* < 0.001, **Figures 1A, B** and **Table 2**). The 3-year OS rate for hyponatremia, hypernatremia and normal sodium subgroups were 33.2, 45.8, and 58.2% respectively. Accordingly, the 3-year DFS rates of the three subgroups were 33.1, 42, and 54.3% respectively. After adjusting other potential confounders including sex, KPS status, tumor location, tumor histology, surgical margin status, vascular invasion, neural invasion, LN dissection number, pTNM stage, NLR, LMR, and adjuvant therapy (**Supplementary Table S1**), sodium status remained significant in predicting OS (hyponatremia, HR [95% CI] = 1.78 [1.2–2.62], *p* = 0.004; hypernatremia, HR [95% CI] = 1.52 [1.1–2.11], *p* = 0.012) and DFS (hyponatremia, HR [95% CI] = 1.52 [1.03–2.23], *p* = 0.036; hypernatremia, HR [95% CI] = 1.45 [1.06–1.99], *p* = 0.021) (**Table 2**).

**Table 2:** Multivariate Cox regression for preoperative serum sodium in whole cohort.

Subgroups	Serum sodium	OS		DFS	
		HR (95% CI)	*p* value	HR (95% CI)	*p* value
All patients ^1^					
	Normal	1(ref.)		1(ref.)	
	Hyponatremia	1.78(1.2–2.62)	0.004	1.52(1.03–2.23)	0.036
	Hypernatremia	1.52(1.1–2.11)	0.012	1.45(1.06–1.99)	0.021
Surgery ^2^					
	Normal	1(ref.)		1(ref.)	
	Hyponatremia	1.6 (1–2.57)	0.05	1.78(1.11–2.84)	0.016
	Hypernatremia	1.49(0.98–2.25)	0.061	1.51(1.01–2.25)	0.043
S+CT/CCRT ^3^					
	Normal	1(ref.)		1(ref.)	
	Hyponatremia	1.74(0.85–3.55)	0.13	1.29(0.63–2.63)	0.483
	Hypernatremia	1.55(0.9–2.65)	0.113	1.42(0.84–2.38)	0.19

CT, chemotherapy; CCRT, concurrent chemoradiotherapy; OS, overall survival; DFS, disease-free survival; HR, hazard ratio; CI, confident interval; ref., reference. Serum sodium levels: Hyponatremia, < 135 mmol/liter; Normal, 135–145 mmol/liter; Hypernatremia, > 145 mmol/liter. ^1^ Multivariate results adjusted by sex, Karnofsky Performance Status, tumor histology, tumor location, tumor differentiation, resection margin, vascular invasion, neural invasion, dissected lymph node number, pTNM stage, NLR, LMR and treatment groups (S + CT/CCRT vs. Surgery). ^2^ Multivariate results adjusted by sex (Female vs. male), KPS score (70 to 80 vs. 90 to 100), tumor histology (adenocarcinoma, neuroendocrine or others vs. squamous carcinoma), tumor differentiation (G2 or G3 vs. G1), resection margin (R1/R2 vs. R0), vascular invasion (Yes vs. No), neural invasion (Yes vs. No), dissected lymph node number (≥ 10 nodes vs. < 10 nodes), pTNM stage (IB–IV vs. IA), NLR and LMR. ^3^ Multivariate results adjusted by sex, tumor histology, tumor differentiation, resection margin, vascular invasion, neural invasion, dissected lymph node number and pTNM stage.

Subgroup analysis showed that the prognostic value of hyponatremia and hypernatremia was confirmed in patients who received surgery alone (**Supplementary Figure S1** and **Table 2**). In the adjuvant CT/CCRT subgroup, however, there was no significant difference in outcome among patients with hyponatremia, hypernatremia and normal serum sodium (**Supplementary Figure S2** and **Table 2**).

### Prognostic Value of Serum Sodium Concentration Within the Reference Range

**Figure 2:**
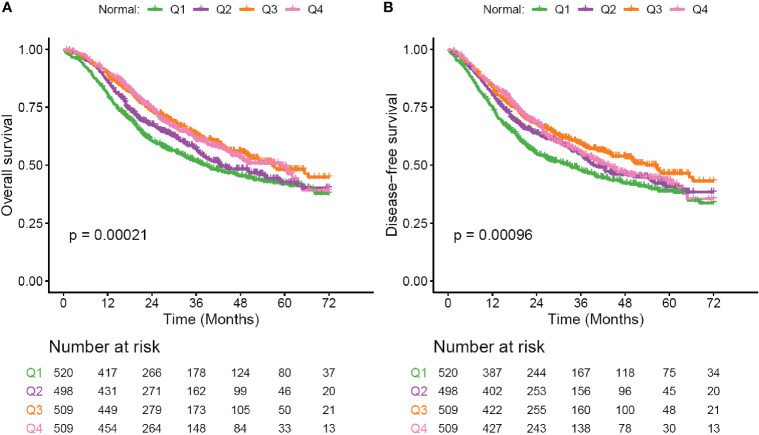
Survival curves for patients with serum sodium within reference range. **(A)** Overall survival (OS) curves for subgroups defined by four ordinal quartiles (Q1-Q4) of normal serum sodium levels. **(B)** Disease-free survival (DFS) curves for subgroups defined by four ordinal quartiles (Q1–Q4) of normal serum sodium levels. Serum sodium subgroups: Q1, 135–139.6 mmol/liter; Q2, 139.6–141.05 mmol/liter; Q3, 141.05–142.4 mmol/liter; Q4, 142.4–145 mmol/liter. Survival curves are generated by using Kaplan-Meier methods. *p* value is calculated by log-rank test.

Although baseline hyponatremia and hypernatremia could predicte poor OS and DFS, the two subgroups only presented 2 and 3.5% of all patients as described above. We thought to investigate whether serum sodium concentration within the reference range could predict outcome. Patients were divided into four ordinal quartile categories (Q1 to Q4), which were significantly associated with patient sex, tumor histology type, pTNM stage and LMR (**Supplementary Table S2**). In Kaplan-Meier analysis, decreased serum sodium concentrations were significantly associated with poor OS and DFS (both *p* < 0.001, **Figures 2A, B**). The 3-year OS rate of patients in Q1 to Q4 subgroups were 51.7, 57.2, 62.9, and 60.8%, respectively. Meanwhile, the 3-year DFS rate were 47.9, 54.8, 59.1, and 55.3% for the four subgroups. Multivariate Cox regression showed that HRs (95% CI) of serum sodium quartiles 2, 3 and 4 compared to the lowest quartile were 0.85 (0.71–1.01), 0.73 (0.61–0.89) and 0.78 (0.64–0.94) for OS (**Table 3**). For DFS, adjusted HRs (95% CI) of serum sodium quartiles 2, 3 and 4 compared to quartile 1 were 0.81 (0.68–0.97), 0.75 (0.63–0.9) and 0.84 (0.7–1.01), respectively (**Table 3**). In addition, the prognostic significance of serum sodium within reference range was observed in the Surgery subgroup (**Supplementary Figure S3** and **Table 3**), but not in patients who received adjuvant CT/CCRT (**Supplementary Figure S4** and **Table 3**).

**Table 3:** Multivariate Cox regression for sequential quartiles of serum sodium within the reference range.

Subgroups	Serum sodium	OS		DFS	
		HR (95% CI)	*p* value	HR (95% CI)	*p* value
All patients ^1^					
	Q1	1(ref.)		1(ref.)	
	Q2	0.85(0.71–1.01)	0.069	0.81(0.68–0.97)	0.02
	Q3	0.73(0.61–0.89)	0.001	0.75(0.63–0.9)	0.002
	Q4	0.78(0.64–0.94)	0.011	0.84(0.7–1.01)	0.066
Surgery ^2^					
	Q1	1(ref.)		1(ref.)	
	Q2	0.79(0.63–1)	0.046	0.77(0.62–0.97)	0.026
	Q3	0.67(0.53–0.85)	< 0.001	0.66(0.53–0.83)	< 0.001
	Q4	0.7(0.55–0.89)	0.003	0.72(0.57–0.9)	0.005
S+CT/CCRT ^3^					
	Q1	1(ref.)		1(ref.)	
	Q2	0.92(0.67–1.25)	0.598	0.88(0.66–1.19)	0.412
	Q3	0.84(0.6–1.18)	0.309	0.93(0.68–1.26)	0.624
	Q4	0.93(0.67–1.3)	0.665	1.07(0.79–1.45)	0.648

CT, chemotherapy; CCRT, concurrent chemoradiotherapy; OS, overall survival; DFS, disease-free survival; HR, hazard ratio; CI, confident interval; ref., reference. Serum sodium levels: Q1, 135–139.6 mmol/liter; Q2, 139.6–141.05 mmol/liter; Q3, 141.05–142.4 mmol/liter; Q4, 142.4–145 mmol/liter. ^1^ Results show multivariate Cox regression adjusted by sex, Karnofsky Performance Status, tumor histology, tumor location, tumor differentiation, resection margin, vascular invasion, neural invasion, dissected lymph node number, pTNM stage, NLR, LMR and treatment groups. ^2-3^ multivariate Cox analysis by using the same confounders as above, except treatment groups.

### Low Serum Sodium Level Predicts Survival Benefit of Adjuvant Therapy

**Figure 3:**
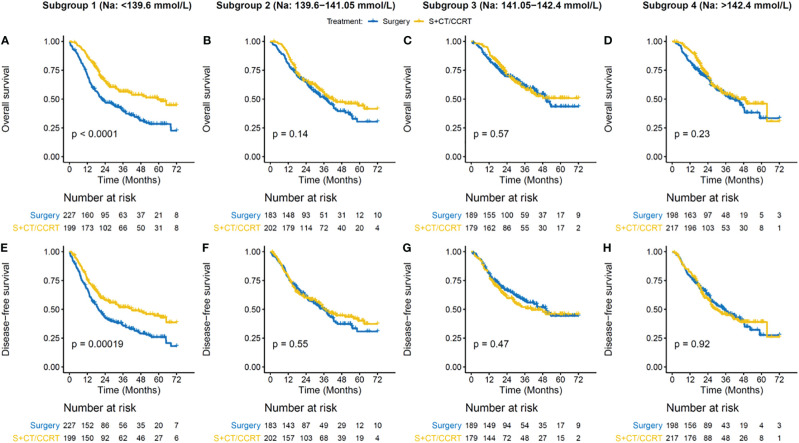
Survival benefit of adjuvant CT/CCRT by serum sodium in the PSM cohort. **(A–D)** Overall survival (OS) curves for treatment groups in different subgroups. **(E–H)** Disease-free survival (DFS) curves for treatment groups in different subgroups. Patient subgroups (each column) were stratified by preoperative serum sodium levels, which are indicated on the top panel. Survival curves are generated by using Kaplan-Meier methods. *p* value is calculated by log-rank test.

The PSM cohort showed much more balanced patient characteristics than those in the initial cohort (**Supplementary Table S3**). By preoperative serum sodium levels, patients were grouped into four subgroups, in which hyponatremia and hypernatremia were combined with Q1 and Q4 respectively. In the lowest serum sodium subgroup (< 139.6 mmol/liter), S + CT/CCRT significantly improved OS (3-year OS rate, 56.6 vs. 40%, *p* < 0.001; adjusted HR [95% CI] = 0.55[0.41–0.73]) and DFS (3-year DFS rate, 51.9 vs. 36.2%, *p* < 0.001; adjusted HR [95% CI] = 0.63[0.48–0.83]) (**Figure 3** and **Table 4**), as compared with Surgery. In other serum sodium subgroups, there was no consistent statistical evidence supporting that S + CT/CCRT could improve OS or DFS as compared with Surgery (**Figure 3** and **Table 4**). Meanwhile, subgroup analysis by chemo regimens demonstrated similar results (**Supplementary Figures S5** and **S6**).

**Table 4:** Cox regression for adjuvant therapy by serum sodium levels.

Sodium levels	Treatment	OS		DFS	
		HR (95% CI)	*p* value	HR (95% CI)	*p* value
Subgroup 1					
	Surgery	1(ref.)		1(ref.)	
	S + CT/CCRT^1^	0.54(0.42–0.71)	< 0.001	0.62(0.48–0.8)	< 0.001
	S + CT/CCRT^2^	0.55(0.41–0.73)	< 0.001	0.63(0.48–0.83)	< 0.001
Subgroup 2					
	Surgery	1(ref.)		1(ref.)	
	S + CT/CCRT^1^	0.81(0.61–1.08)	0.145	0.92(0.7–1.21)	0.548
	S + CT/CCRT^2^	0.68(0.5–0.92)	0.012	0.81(0.6–1.08)	0.155
Subgroup 3					
	Surgery	1(ref.)		1(ref.)	
	S + CT/CCRT^1^	0.91(0.65–1.26)	0.569	1.12(0.82–1.53)	0.473
	S + CT/CCRT^2^	0.87(0.62–1.24)	0.447	1.15(0.83–1.58)	0.411
Subgroup 4					
	Surgery	1(ref.)		1(ref.)	
	S + CT/CCRT^1^	0.83(0.62–1.12)	0.233	1.01(0.77–1.34)	0.915
	S + CT/CCRT^2^	0.8(0.58–1.1)	0.164	1.01(0.76–1.35)	0.927

CT, chemotherapy; CCRT, concurrent chemoradiotherapy; OS, overall survival; DFS, disease-free survival; HR, hazard ratio; CI, confident interval; ref., reference. Serum Na levels: subgroup 1, < 139.6 mmol/liter; subgroup 2, 139.6–141.05 mmol/liter; subgroup 3, 141.05–142.4 mmol/liter; subgroup 4, > 142.4 mmol/liter. ^1^Results show univariate Cox regression. ^2^Results show multivariate Cox regression adjusted by sex, age, Karnofsky Performance Status, tumor histology, tumor location, tumor differentiation, resection margin, vascular invasion, neural invasion, dissected lymph node number, pTNM stage, NLR and LMR.

Stratified analysis was performed by traditional high-risk factors, including pT3-4, nodal involvement and/or positive surgical margin. Survival advantage of adjuvant therapy was observed in patients at high-risk, but absent in those at low-risk (**Supplementary Figure S7** and**Tables S4** and **S5**). When sodium subgroups were taken into consideration, patients with Na < 139.6 mmol/liter but with traditional low-risk factors demonstrated a trend toward benefit from adjuvant CT/CCRT (**Supplementary Figure S8** and **Table S5**). For high-risk patients, S + CT/CCRT seemed not to be beneficial in those with Na from 141.05 to 142.4 mmol/liter (**Supplementary Figure S9** and **Table S4**).

### Serum Sodium Concentration Correlates With Inflammatory Response

**Figure 4:**
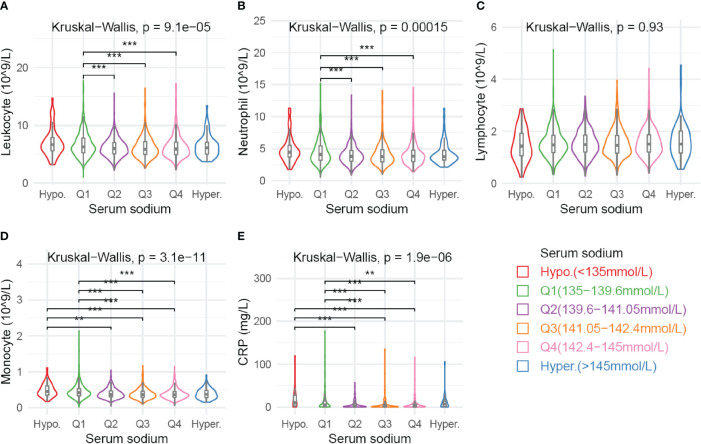
Association of various blood parameters with serum sodium concentration. Distribution of cell counts of leukocyte **(A)**, neutrophil **(B)**, lymphcyte **(C)**, and monocyte **(D)** are presented across subgroups defined by sodium levels. Serum CRP levels **(E)** are available for 664 patients. Comparison among all subgroups was performed by Kruskal-Wallis test. Significance between each pair of subgroups was estimated by Mann-Whitney test. Multiple tests were adjusted by using Bonferroni method. Adjusted *p* values: *p < 0.05; **p < 0.01; ***p < 0.001.

The peripheral number of total leukocytes and neutrophils were significantly higher in Q1 than in Q2-Q4 subgroups (**Figure 4**). Monocytes counts and CRP levels were significantly elevated in subgroups with low serum sodium (hyponatremia or Q1) as compared with other sodium subgroups. However, there were no significant differences on lymphocytes numbers among sodium subgroups. Notably, the hypernatremia subgroup also showed a trend toward elevated serum CRP levels as compared with Q2-Q4 subgroups (**Figure 4**). Meanwhile, similar results were observed in the PSM cohort (**Supplementary Figure S10**).

## Discussion

In this study, we retrospectively evaluated the prognostic value of preoperative serum sodium in patients with EC. The incidences of hyponatremia and hypernatremia in treatment-naïve patients were 2 and 3.5% respectively. Hyponatremic and hypernatremic patients had 78 and 52% increase in the risk of death, as well as 52 and 45% increase in the risk of disease recurrence. Meanwhile, we found that patients with lower serum sodium concentrations (Na < 139 mmol/liter) would more likely get benefit from adjuvant CT/CCRT, compared with the other sodium subgroups. Furthermore, preoperative serum sodium concentration was inversely correlated with leukocyte, neutrophil and monocyte counts. To the best of our knowledge, this is the largest study to investigate the prognostic and predictive value of preoperative serum sodium in patients with EC.

Hyponatremia occurred at a relative high frequency (16–59%) in EC patients who previously received chemotherapy (17–22). However, we found that the incidence of hyponatremia in treatment-naïve EC patients was only 2%. The difference may be explained by the fact that chemo-reagents could induce hyponatremia in cancer patients (10). The difference could also be explained to genetic polymorphisms in patients, which could influence hyponatremia in ESCC (24). We found that preoperative hyponatremia was significantly associated with poor postoperative prognosis, although the significance was not achieved in patients who received adjuvant therapy (21).

Previous reports showed that hypernatremia was associated with higher mortality in hospitalized patients with diverse types of cancer (25, 26). Our results confirmed that preoperative hypernatremia was significantly correlated with poor outcome in EC patients. The increased serum CRP in hypernatremic patients may be associated with increased inflammatory responses in cancer patients (27). In addition, we found that the serum sodium concentrations within the reference range (135–145 mmol/liter) were inversely correlated with outcome in EC patients. However, the association between sodium concentration and survival rate did not exactly fit a linear model, since the Q4 subgroup showed similar or even worse prognosis compared with the Q3 subgroup. This was inconsistent with a previous report which showed that each 3 mmol/liter decrease in serum sodium concentration was associated with 19% increase in risk of death for metastatic renal cell carcinoma (16). The difference may be partially explained by different cancer types. Meanwhile, the poor prognosis of hyponatremia and hypernatremia suggested that both low- and high-level of serum sodium may be unfavorable for patients with ECs. This could be explained by the fact that hyponatremia or hypernatremia is associated with dysfunction of sodium absorption or excretion, as well as an increase or decrease in total body water. These electrolyte imbalances may be highly correlated with complications of patients with advanced cancer such as renal dysfunction, dehydration or volume overload due to heart and liver failure (25, 26).

Little is known about biomarkers which could predict benefit of adjuvant therapy in patients with EC. A few studies demonstrated that adjuvant chemotherapy or chemoradiotherapy after curative surgical resection could decrease recurrence and prolong survival in patients with clinical high-risk factors (pT3-4, pN+, or R1/2 resection) (3–9). Two retrospective studies based on PSM showed that postoperative chemoradiotherapy could improve OS and DFS in unselected patients with ESCC (28, 29). By using PSM analysis, we confirmed that postoperative chemotherapy or chemoradiotherapy were associated with better OS and DFS compared with surgery alone. However, the benefit of adjuvant therapy remarkably differed in subgroups stratified by preoperative serum sodium concentration. Patients with low sodium concentrations (Na < 139 mmol/liter) were more likely beneficial from adjuvant therapy. In contrast, there was no significance of difference in outcome between adjuvant therapy and surgery alone in Q3 subgroup (Na: 141.1–143 mmol/liter), even for patients with traditional high-risk factors. These findings suggested that preoperative sodium concentration, as a compensation of clinical high-risk factors, may serve as a predictive marker for adjuvant therapy in EC patients.

The mechanism underlying the development of hyponatremia in EC patients remains unclear. The syndrome of inappropriate antidiuretic hormone (SIADH) could be induced by chemotherapy, since several cases were diagnosed with hyponatremia and SIADH after receiving chemotherapy (30–35). However, SIADH in treatment-naïve EC patients was poorly known. Another possible factor that influences serum sodium level is systemic inflammatory response. We found that the inflammatory parameters including leukocytes, neutrophils, monocytes and CRP levels were significantly elevated in patients with low sodium levels. Consistent with our results, Shimada et al. (21) reported that baseline white blood cell count was a risk factor for hyponatremia in EC patients. Meanwhile, the inflammation associated hyponatremia also occurred in non-malignant diseases, in which hyponatremia was correlated with neutrophil counts, CRP, IL-1β, and IL-6 levels (36). Proinflammatory cytokines IL-1β and IL-6 could promote neurons to secret ADH, thereby decreasing serum sodium levels (37, 38). Meanwhile, cell swelling stimulated by low osmolality might induce inflammasome activation in macrophages, which promote inflammatory response in return (39). Therefore, a vicious cycle was proposed between inflammation and hyponatremia (40). However, the causal relationship between hyponatremia and inflammation in EC patients remains unknown and requires further investigation.

In summary, the large-scale retrospective study demonstrated that decreased preoperative serum sodium level was correlated with poor prognosis in EC patients. Low serum sodium may serve as a predictive marker for postoperative adjuvant therapy. Meanwhile, serum sodium level was inversely associated with systemic inflammatory response. These results need to be further verified in prospective, randomized controlled trials.

## Data Availability Statement

The original contributions presented in the study are included in the article/supplementary material; further inquiries can be directed to the corresponding author.

## Ethics Statement

The studies involving human participants were reviewed and approved by Institutional Ethics Committee of Sichuan Cancer Hospital. The patients/participants provided their written informed consent to participate in this study.

## Author Contributions

YH, JL, and BC conceptualized the study. WD, YWa, LW, YWe, and QF performed the data curation. BC performed the formal analysis. QW LP, JL, and BC acquired the funding. QW and BC conducted the investigation. QW and LP developed the methodology. QW and TX conducted the project administration. TL and QL provided the resources. BC provided the Software. LP and TX supervised the study. LP, WD, YW, LW, YWa, and QF validated the study. BC conducted the visualization. QW and BC wrote the original draft. YH, TL, QL and JL wrote, reviewed, and edited the manuscript. All authors contributed to the article and approved the submitted version.

## Funding

This work was supported by National Natural Science Foundation of China (grant number 81602731), Ministry of Science and Technology of China (grant number 2017YFC0113101), Department of Science and Technology of Sichuan Province (grant numbers 2019YFS0378 and 2018JY0277), the Cancer Research Foundation of China Anti-cancer Association for Young Scientists (grant number CAYC18A33), and CSCO-Genecast Oncology Research Found (grant number Y-2019Genecast-041).

## Conflict of Interest

The authors declare that the research was conducted in the absence of any commercial or financial relationships that could be construed as a potential conflict of interest.
