# Supplementary material for: Preoperative Serum Sodium Level as a Prognostic and Predictive Biomarker for Adjuvant Therapy in Esophageal Cancer
Source: Front Oncol. 2021 Jan 21;10:555714. doi: 10.3389/fonc.2020.555714 (PMC7858663; doi:10.3389/fonc.2020.555714)
Supplement: Supplementary file 1 [file DataSheet_1.docx]

Supplementary Figure S1. Survival analysis for preoperative serum sodium in patients who received surgery alone. A, analysis of OS. B, analysis of DFS.

Supplementary Figure S2. Survival analysis for preoperative serum sodium in patients who received adjuvant therapy following surgery. A, analysis of OS. B, analysis of DFS.

Supplementary Figure S3. Survival analysis serum sodium within reference range in patients who received surgery alone. A, analysis of OS. B, analysis of DFS.

Supplementary Figure S4. Survival analysis serum sodium within reference range in patients who received adjuvant therapy following surgery. A, analysis of OS. B, analysis of DFS.

Supplementary Figure S5. Survival benefit of adjuvant CT/CCRT stratified by chemo regimens, in sub-cohort of patients with serum sodium < 139.6 mmol/L.

Supplementary Figure S6. Survival benefit of adjuvant CT/CCRT stratified by chemo regimens, in sub-cohort of patients with serum sodium ≥ 139.6- mmol/L.

Supplementary Figure S7. Survival benefit of adjuvant CT/CCRT in patients at low-risk (A-B) and high-risk (C-D), respectively.

Supplementary Figure S8. Survival benefit of adjuvant CT/CCRT stratified by serum sodium, in sub-cohort of patients at low-risk.

Supplementary Figure S9. Survival benefit of adjuvant CT/CCRT stratified by serum sodium, in sub-cohort of patients at high-risk.

Supplementary Figure S10. Association of various blood parameters with serum sodium concentration in the PSM cohort.
